# Supplementary material for: Field validation of multiple species distribution models shows variation in performance for predicting Aedes albopictus distributions at the invasion edge
Source: Parasit Vectors. 2025 Nov 25;18:488. doi: 10.1186/s13071-025-07117-y (PMC12648814; doi:10.1186/s13071-025-07117-y)

Supplementary Information

**S1 Figure.** Map of Suffolk County Long Island, New York, U.S.A., depicting the number of years (one mosquito season per year) that each site was sampled. Points represent sites and are colored by the number of years that the individual site was sampled. Suffolk County shape file from NYS GIS Civil Boundaries Program (https://gis.ny.gov/civil-boundaries).


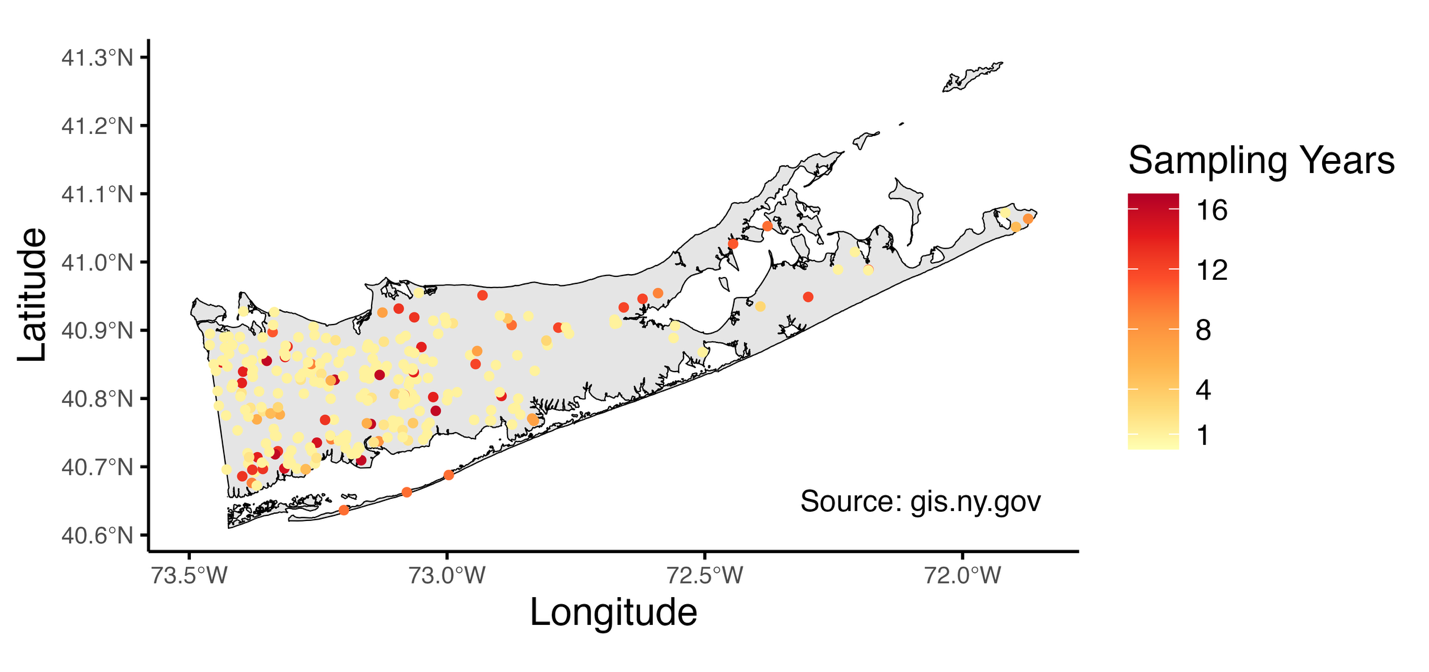


**S2 Figure.** Map of Suffolk County, New York, U.S.A., depicting the invasion of *Aedes albopictus* from 2008 to 2023. Sites where at least one female *Ae. albopictus* had been caught are pictured as points. Point color is based on the year *Ae. albopictus* was first detected. Sites that have never had *Ae. albopictus* are not pictured. Suffolk County shape file from NYS GIS Civil Boundaries Program (https://gis.ny.gov/civil-boundaries).


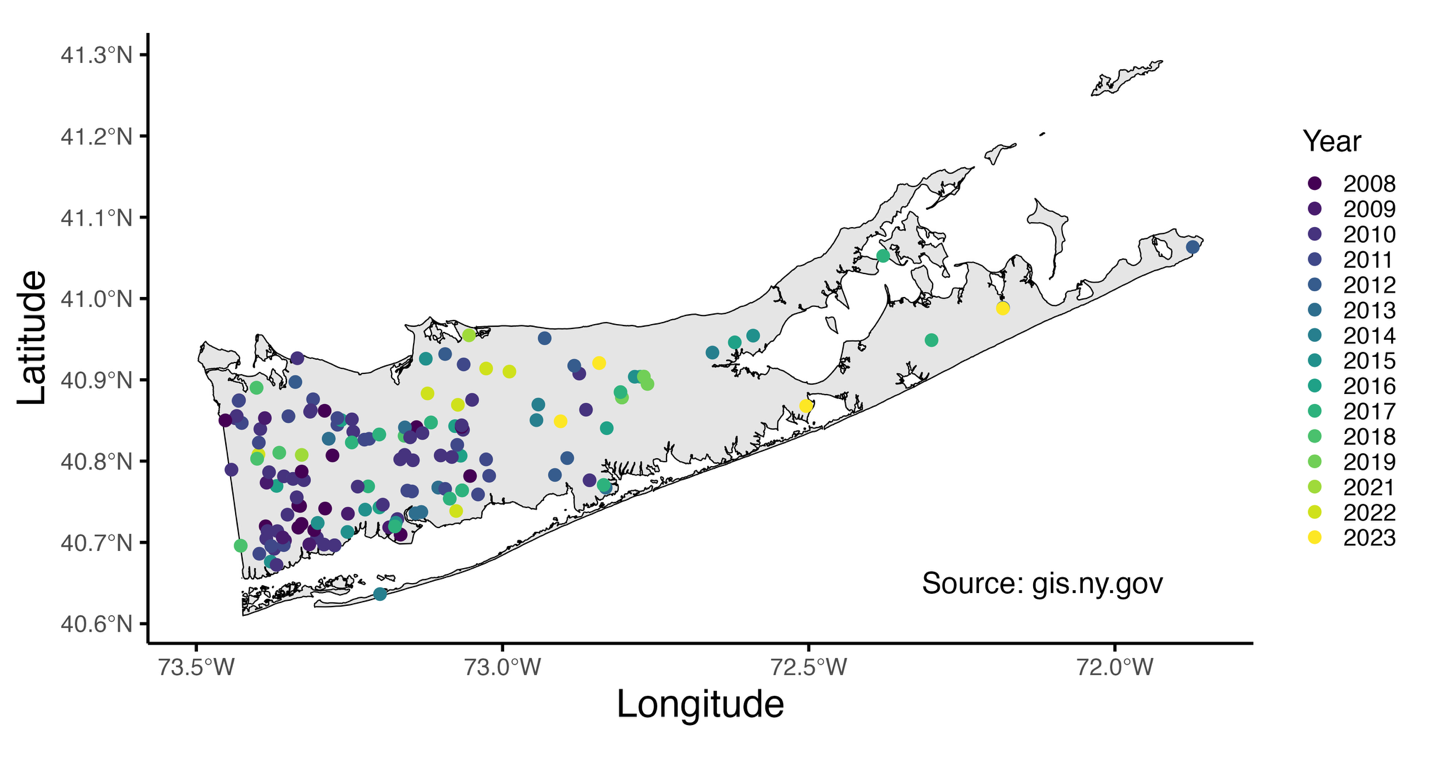


**S3 Figure.** Modified expanding window training approach to explore how the addition of more training data improves each SDM’s ability to predict late-stage invasion distributions (testing data). Each training iteration included an additional year of training data chronologically.


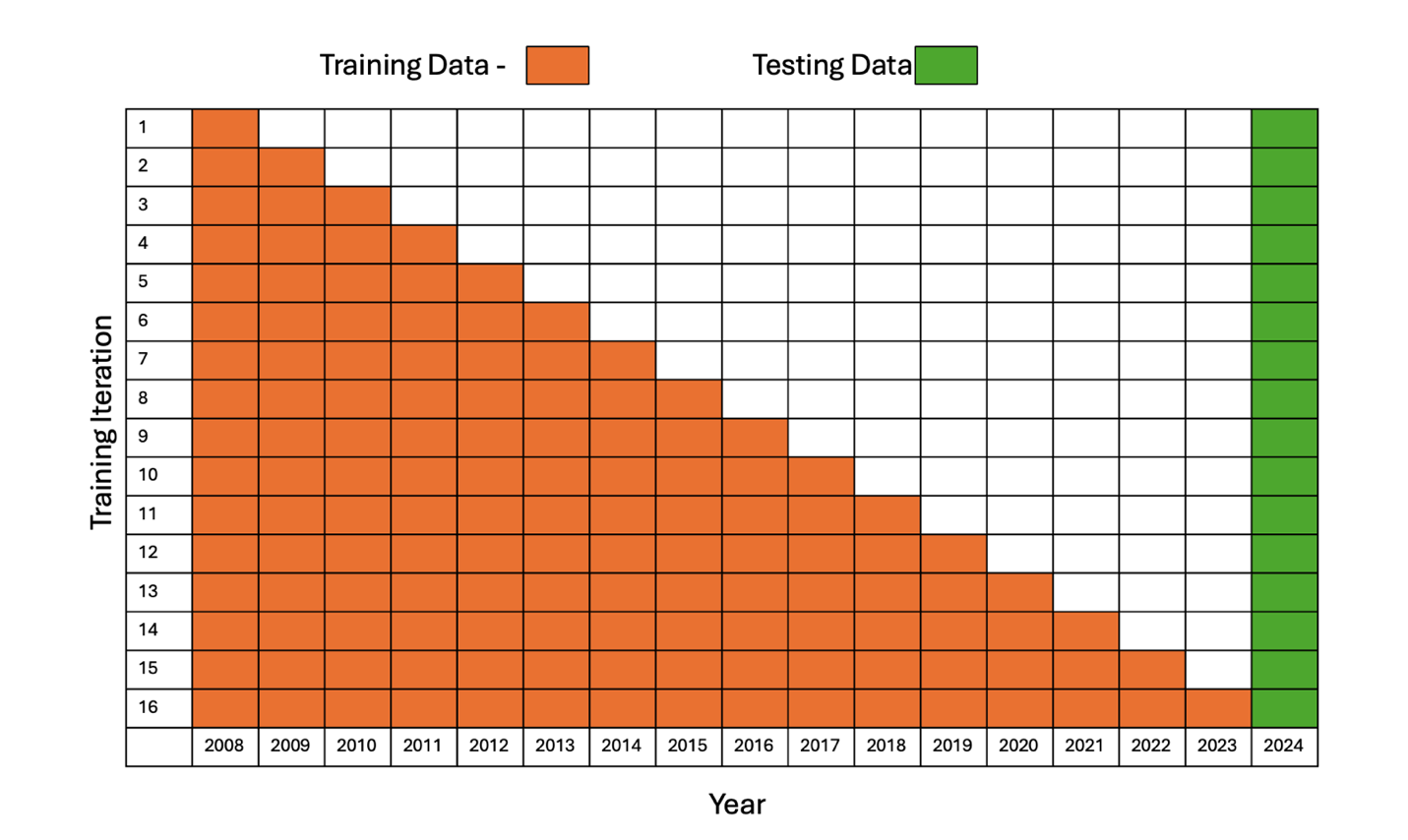


**S4 Figure.** Graphs display variation in a) % impervious surface, b) land cover, c) EVI, d) day surface temperature, and e) night surface temperature in Suffolk County, NY. These values are based on remote-sensed data averaged across May - October 2023. Color represents different values for each environmental variable. Temperature is represented in °C. Suffolk County shape file from NYS GIS Civil Boundaries Program (https://gis.ny.gov/civil-boundaries).


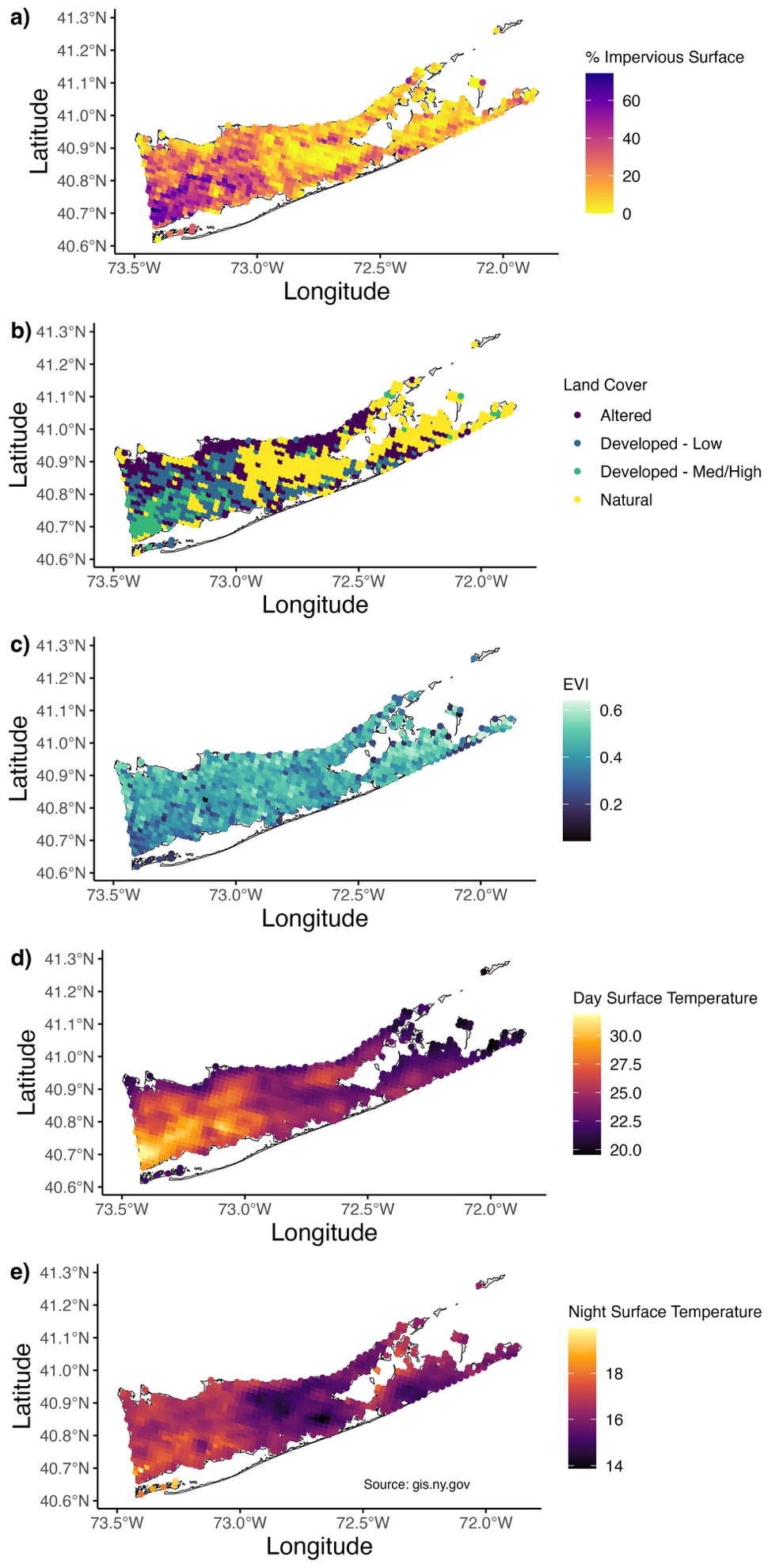


**S5 Figure.** Graphs represent receiver-operator curves generated from random sampling cross-validation. Each graph represents a different model. Each colored line represents a different iteration (different 20% testing and 80% training datasets, randomly selected) of cross-validation with bold black lines representing averages for testing (solid) and training (dashed) data. Text bubbles on each graph summarize the average AUC values for all training dataset iterations and all testing dataset iterations. As AUC gets closer to one, the model is considered to perform better. An AUC of one would indicate a model that can perfectly identify true presences and true absences.


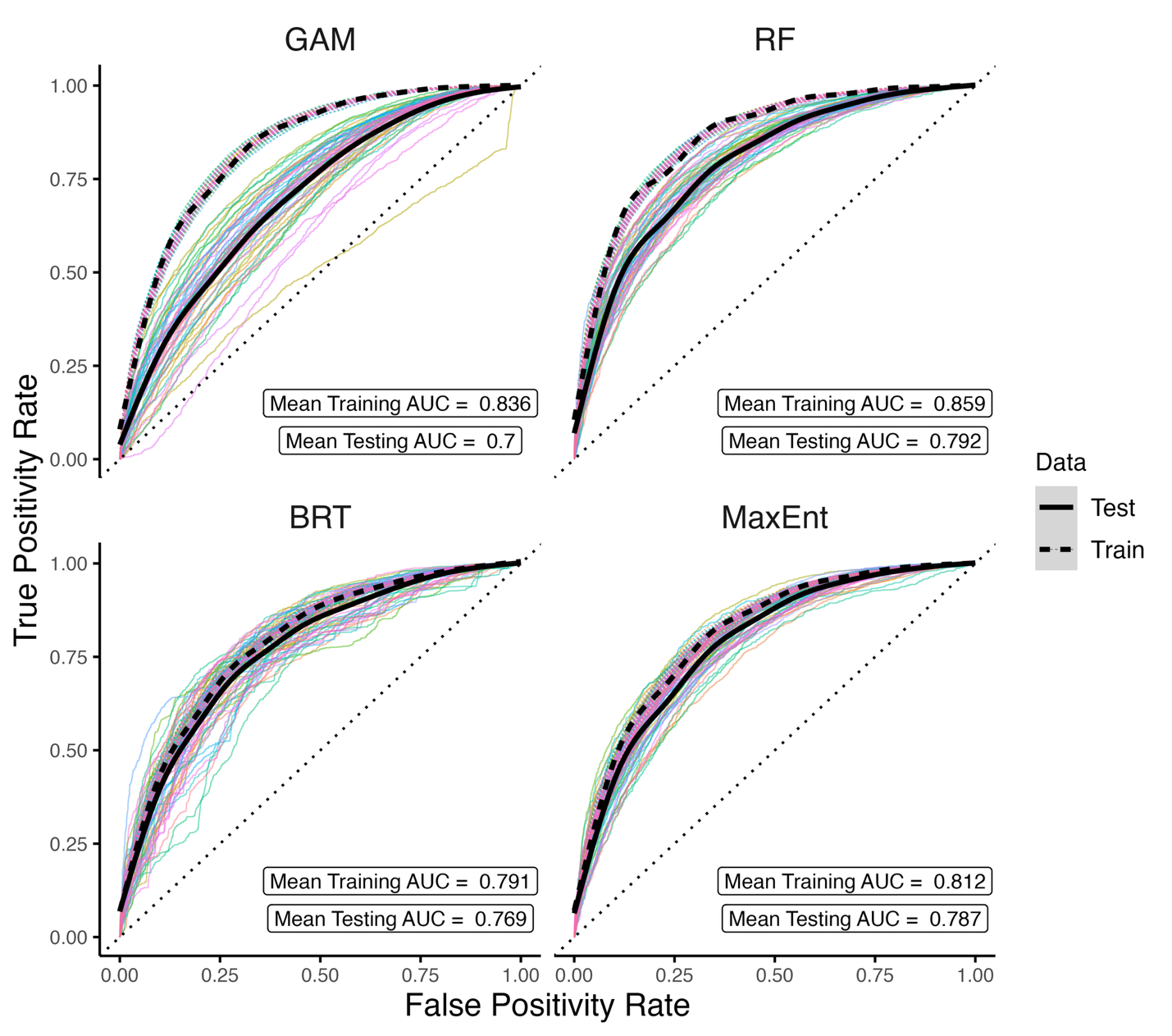


**S6 Figure.** Graph represents receiver-operator curves generated from random sampling cross-validation for the mechanistic SDM based on temperature-dependent population density. Each colored line represents a different iteration (different 20% testing and 80% training datasets, randomly selected) of cross-validation with bold black lines representing averages for testing (solid) and training (dashed) data. Text bubbles summarize the average AUC values for all training dataset iterations and all testing dataset iterations. As AUC gets closer to one, the model is considered to perform better. An AUC of one would indicate a model that can perfectly identify true presences and true absences. AUCs close to 0.5 indicate that the model is no better at predicting presence or absence than a coin toss.


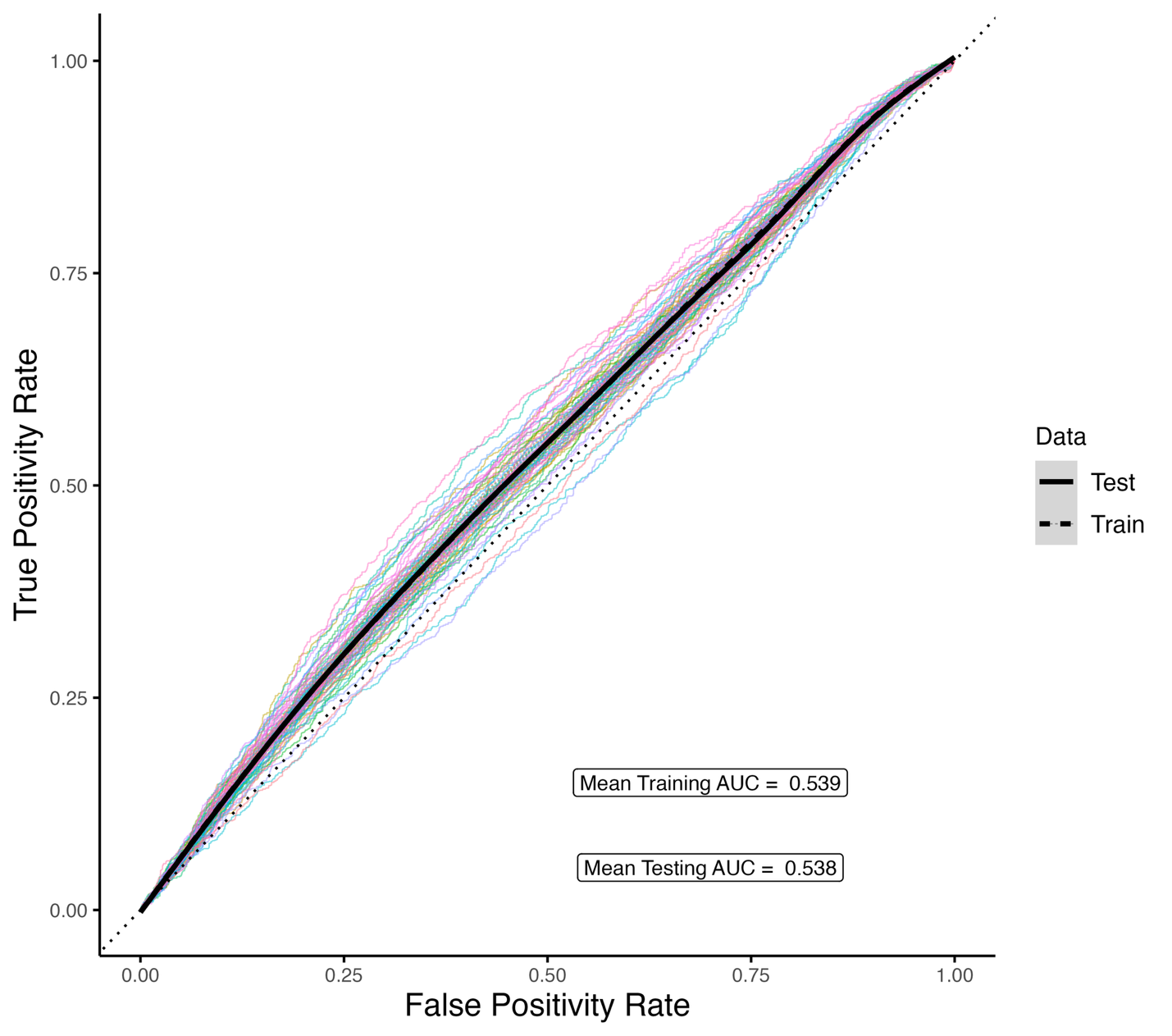


**S7 Figure.** Graphs represent receiver-operator curves generated from township sampling cross-validation. Each graph represents a different model. Each colored line represents a different iteration (different 20% testing and 80% training datasets, randomly selected) of cross-validation with bold black lines representing averages for testing (solid) and training (dashed) data. Text bubbles on each graph summarize the average AUC values for all training dataset iterations and all testing dataset iterations. As AUC gets closer to one, the model is considered to perform better. An AUC of one would indicate a model that can perfectly identify true presences and true absences.


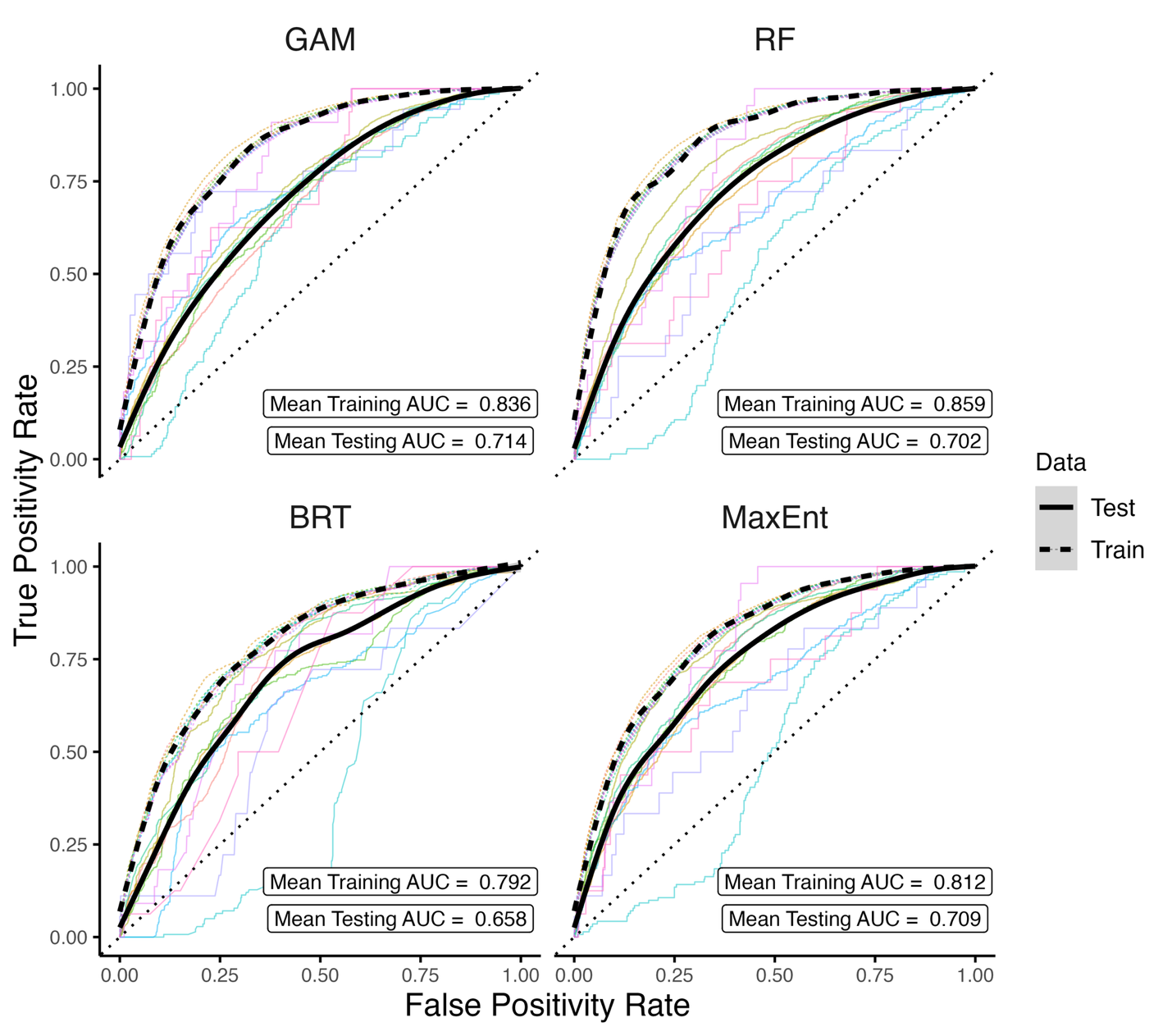


**S8 Figure.** Partial dependence plots for seven covariates of interest: a) impervious surface, b) EVI, c) daytime land surface temperature, d) nighttime land surface temperature, e) Month, f) Year, and g) Land cover type that predict habitat suitability for *Aedes albopictus* in Suffolk County, New York, U.S.A. Points are colored by modeling method (BRT = Boosted Regression Tree, navy; GAM = Generalized Additive Model, blue; MaxEnt = Maximum Entropy, green; and RF = Random Forest, yellow). Suitability on the y-axis represents habitat suitability for *Aedes albopictus*.


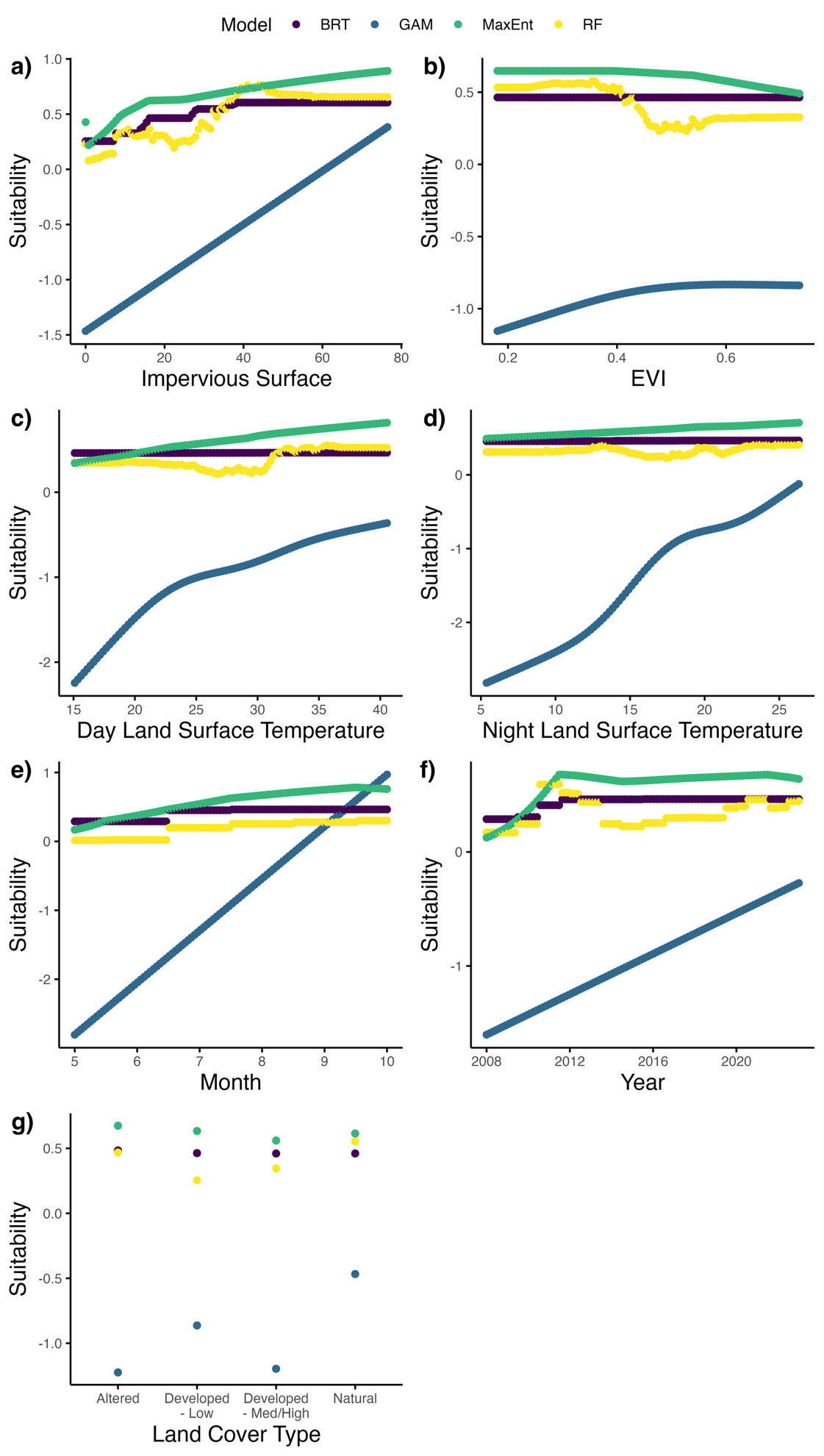


**S9 Figure.** Graph represents total disagreement among our four species distribution models (Boosted Regression Tree, Generalized Additive Models, Maximum Entropy, and Random Forest) in predicting *Aedes albopictus* presence from July – September 2024 across different land use categories of Suffolk County, New York, U.S.A. Land use categories were determined from NLCD categories: natural (forested, herbaceous, water, wetlands, shrub, barren), altered (open – developed, cultivated), Developed – Low, Developed – Med/High (Developed – Med, Developed High). Higher values indicate higher disagreement among model predictions. Bars represent average disagreement. Letters indicate significant differences (α=0.05) calculated from an ANOVA followed by a Tukey post-hoc analysis.


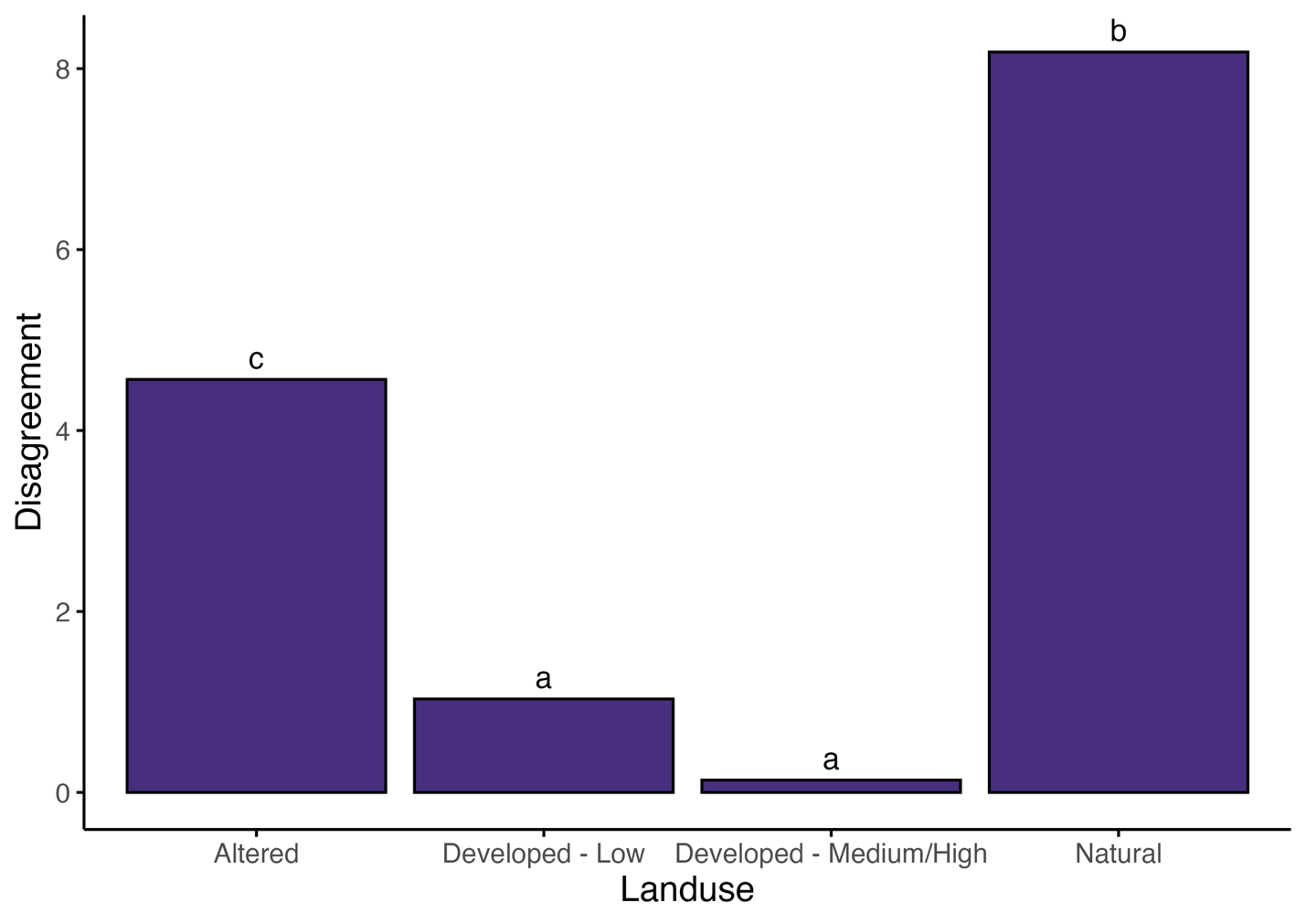

Supplement: Supplementary file 1 — Additional file1 [file 13071_2025_7117_MOESM1_ESM.docx]
